# Aortic Coarctation

**DOI:** 10.1055/s-0040-1701522

**Published:** 2020-07-31

**Authors:** Umberto G. Rossi, Anna Maria Ierardi, Gianpaolo Carrafiello, Maurizio Cariati

**Affiliations:** 1 Department of Diagnostic Imaging, Interventional Radiology Unit, Ente Ospedaliero Galliera Hospital, Genova, Italy; 2 Department of Diagnostic and Therapeutic Advanced Technology, Diagnostic and Interventional Radiology Unit, Azienda Socio Sanitaria Territoriale Santi Paolo and Carlo Hospital, Milano, Italy; 3 Department of Services and Preventive Medicine, Radiology Unit, Ca' Granda Fondation, Maggiore Policlinic Hospital, University of Milan, Milano, Italy

**Keywords:** aorta, coarctation, vascular pathology, computed tomography, imaging, aging

## Abstract

We report a case of a 45-year-old male suffering from arterial hypertension who was found to have an aortic coarctation with marked hypertrophic compensatory collateral arterial circulation. Although coarctation is relatively rare, this must be included in the differential diagnosis in patients with arterial hypertension with a positive gradient between upper and lower limbs.

A 45-year-old male was evaluated for arterial hypertension. A pressure gradient (18 mm Hg) between upper and lower limbs was noted. Among the various diagnostic examinations, the patient underwent multidetector computed tomography (MD-CT) angiography. This showed an aortic coarctation at the level of isthmus proximal third of thoracic aorta with significant hypertrophic compensatory collateral arterial circulation ( Fig. 1 ). At the time of diagnosis, endovascular or open surgery treatment were offered to the patient, but he refused all intervention. Therefore, the patient was started on life-long antihypertensive medical treatment with clinical and imaging follow-up.

**Fig. 1:**
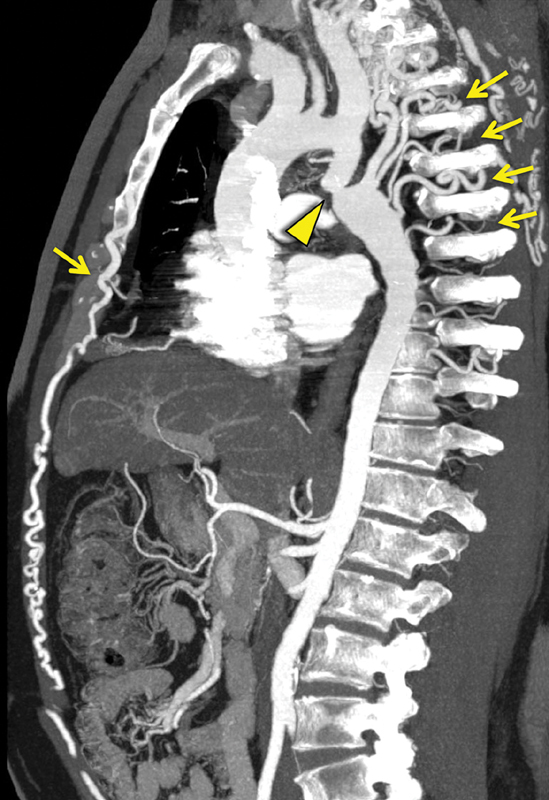
Multidetector computed tomography angiography sagittal reconstruction demonstrating the presence of aortic coarctation at the level of isthmus proximal third of thoracic aorta (arrowhead) with hypertrophic compensatory collateral arterial circulation from the intercostal and mammary arteries (arrows).

Aortic coarctation in adults is generally recognized via systemic arterial hypertension associated with a pressure gradient between the upper and lower extremities. 1 Precise vascular imaging (MD-CT or magnetic resonance imaging) is mandatory for a complete evaluation of the thoracic aorta, its branches, and possible collateral vessels. 2 3 Criteria for invasive treatment in adult patients include translesional pressure gradient (>20 mm Hg) and/or evidence of significant collateral vessels. 1 4 5 6 Choice between open surgery versus percutaneous endovascular treatment should be determined by a multidisciplinary team specialists (surgeons, interventional radiologists, and cardiologists). 1 7 Finally, all patients affected by aortic coarctation require a life-long treatment of arterial pressure and close follow-up (clinical and imaging).
